# Successful treatment of giant abdominal aortic aneurysm with chronic contained rupture diagnosed at the onset of acute retrograde type A aortic dissection: A case report

**DOI:** 10.1016/j.ijscr.2024.109635

**Published:** 2024-04-19

**Authors:** Takafumi Akai, Shintarou Ninomiya, Takanori Kaneko, Takatoshi Furuya

**Affiliations:** Department of Vascular Surgery, Asahi General Hospital, Japan

**Keywords:** Abdominal aortic aneurysm, Aortic dissection, Chronic contained rupture, Tailor-made tapering graft, Case report

## Abstract

**Introduction:**

It is rare for two critical diseases, namely a giant abdominal aortic aneurysm (AAA) and acute type A aortic dissection (TAAD), to be detected simultaneously, and in such instances, management is extremely difficult.

**Presentation of case:**

A 64-year-old man who presented to our hospital with a chief complaint of sudden back pain and vomiting was diagnosed with acute retrograde TAAD and a giant AAA with chronic contained rupture (CCR) via computed tomography. We initially managed the acute TAAD conservatively and subsequently performed laparotomy for the AAA 3 months later. During open surgery, we performed vascular reconstruction using a tailor-made tapering graft.

**Discussion:**

Emergency surgery is recommended for AAA with CCR or retrograde TAAD with a patent false lumen, and the prognosis of conservative treatments for these cases is currently unknown. However, concurrent surgery for this condition is extremely invasive. Fortunately, the patient in this case survived the acute phase, and laparotomy for the AAA could be safely performed during the chronic phase of the TAAD.

**Conclusion:**

We successfully treated a giant AAA with CCR by selecting the appropriate surgical timing and method. In cases of combined CCR of a giant AAA and retrograde TAAD, conservative management may be attempted to convert the acute dissection to a chronic one, thereby allowing elective repair of the AAA.

## Highlights

• TAAD and giant AAA present unique management challenges.
• Conservative TAAD management, followed by AAA laparotomy, proved successful.
• Safe laparotomy performed during TAAD chronic phase, ensuring patient survival
• Vascular reconstruction utilized tailor-made graft during open surgery.

## 1 Introduction

Although a definitive definition for a giant abdominal aortic aneurysm (AAA) is lacking, an AAA is considered giant when its transverse diameter is >10 cm [1], with other sources [2,3] considering higher diameter cut-offs of >11 or 13 cm. Giant AAAs are rare owing to the recommendation of repair once a size of 5.0–5.5 cm is reached and a high rate of rupture when they become >6 cm in size. The risk of AAA rupture is 14 % per year when the size is >6 cm, which increases to 30–50 % per year when the size is >8 cm [4]. When such a dangerous giant AAA is detected together with another life-threatening disease, such as acute type A aortic dissection (TAAD), management becomes difficult. There are few reports on synchronous TAAD and giant AAA; moreover, synchronous TAAD and giant AAA with chronic contained rupture (CCR) has not been reported. Xu et al. [5] reported a concomitant surgical case, although the necessity of performing concomitant surgery remains debatable [6]. Hu et al. [7] reported short-interval staged surgical treatment, which resulted in a good outcome. Xiang et al. [8] reported a case of acute type B aortic dissection with concomitant retrograde type A intramural hematoma and a giant AAA treated by combined thoracic endovascular aortic repair and open surgery for the AAA. Performing one-stage surgery for concurrent TAAD and giant AAA involves a highly extensive, rigorous procedure that carries significant risk for the patient, while a two-stage operation risks the aneurysm for potential rupture. Herein, we report our experience in treating a patient with giant AAA and CCR diagnosed at the onset of acute retrograde TAAD. This case has been reported in line with the SCARE 2023 Criteria [9].

## 2 Presentation of case

A 64-year-old man presented to our hospital with a chief complaint of sudden back pain and vomiting. He had a medical history of hypertension and chronic atrial fibrillation, and a 50-year history of smoking. His heart rate was 66/min and blood pressure was 144/96 mmHg, with no difference between the right and left sides, and bilateral dorsalis pedis artery pulses were palpable. Computed tomography (CT) revealed acute aortic dissection, as well as a giant AAA. He had been aware of a pulsating mass in his abdomen for approximately 5 years but had not visited a medical institution.

**Fig. 1:**
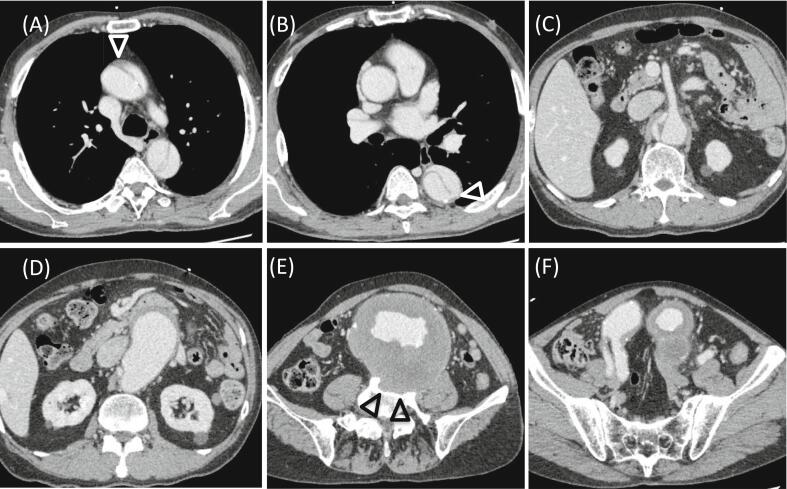
Computed tomography (CT) images on initial admission. (A) The aortic dissection extends from the ascending aorta, and the false lumen is patent. Thrombosis is observed in a small portion of the false lumen of the ascending aorta (arrowhead). (B) The main entry is at the descending aorta (arrowhead). (C) The aortic dissection is noted at the level of the superior mesenteric artery and extends to the vicinity of the right renal artery. (D) The abdominal aortic aneurysm (AAA) has a huge reverse tapering neck. (E) The AAA measures 11.5 × 9.8 cm. Part of the vertebral body is destroyed (arrowheads). (F) The aneurysm extends to the common iliac arteries bilaterally.

Although the aortic dissection on CT extended from the ascending aorta to the vicinity of the right renal artery, the main entry was at the distal side of the left subclavian artery. Therefore, we diagnosed the patient with retrograde TAAD (Fig. 1A, B, C). There was no entry in the ascending arch, and thrombosis was observed in a small portion of the false lumen of the ascending aorta, which had a diameter of 3.9 cm. The AAA was 11.5 × 9.8 cm in size, the neck of the AAA was dilated with reverse tapering, and the aneurysm extended to the common iliac arteries bilaterally. The aortic diameters at the level of the left renal, right common iliac, and left common iliac arteries were 3.7 cm, 3.4 cm, and 4.7 cm, respectively. Part of the vertebral body was destroyed, which was considered evidence of chronic rupture. The walls of the aortic aneurysm in other areas were thickened (Fig. 1D, E, F). Blood tests showed an increased D-dimer level (41.2 μg/ml), while hemoglobin (13.3 mg/dl) and creatinine (0.82 mg/dl) levels were within normal ranges.

**Fig. 2:**
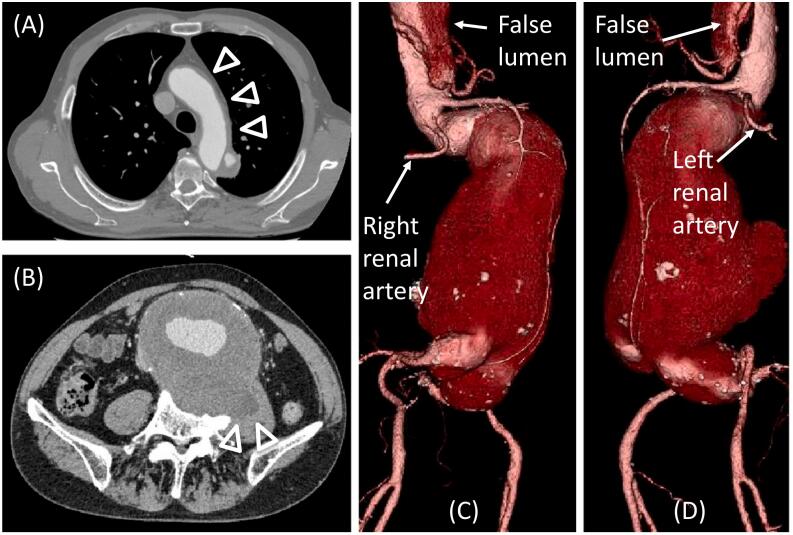
Preoperative enhanced computed tomography (CT) images. (A) False lumen from the ascending aorta to the aortic arch is occluded by thrombus (arrowheads). (B) Slight progression in the chronic rupture (arrowheads) compared to that at the time of initial admission is observed. Volume rendering images of the CT angiography on the right anterior oblique position (C) and left anterior oblique position (D) show almost no enlargement in the size of the AAA. The false lumen was patent at the vicinity of the right renal artery.

After consultation with the cardiovascular surgery team, the patient was informed of the risk of death associated with conservative treatment, but he refused surgery. Hence, we proceeded to treat the aortic dissection conservatively. The patient was in a state of acute dissection, and considering the surgical risks, we decided to perform elective surgery for the AAA. Medical therapy with tight blood pressure control and analgesic treatment was administered. Oxygenation was poor, but mechanical ventilation was not required. With conservative treatment, the aortic dissection was occluded by a thrombus from the ascending aorta to the aortic arch, and no enlargement of the descending aortic diameter was observed. The patient was discharged home 34 days after admission. As the AAA was juxtarenal, we decided to perform open surgery 3 months after the acute dissection. On preoperative enhanced CT, the aortic dissection was still occluded by a thrombus from the ascending aorta to the aortic arch (Fig. 2A). Although there was slight progression in the state of chronic rupture, the AAA measured 11.4 × 10.0 cm, with almost no enlargement from the time of initial admission (Fig. 2B, C, D).

**Fig. 3:**
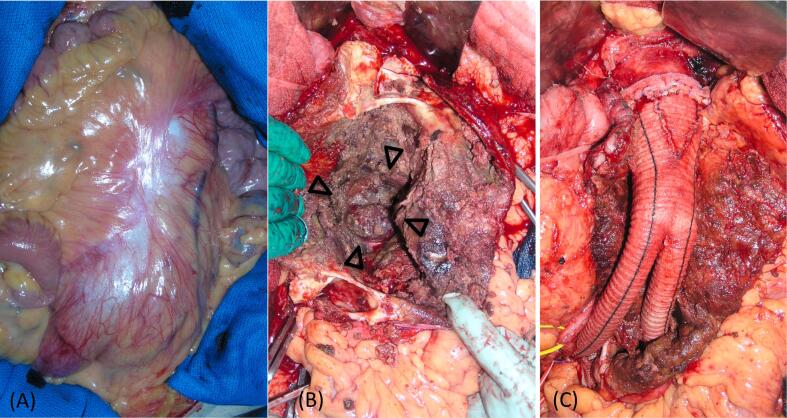
Intraoperative images. (A) The front surface of the aneurysm appears white and shiny. (B) The vertebral body is exposed on the dorsal side of the aneurysm (arrowheads). (C) Vascular reconstruction is performed using a tailor-made tapering graft.

Surgery was performed under general anesthesia. During laparotomy, the front surface of the aneurysm appeared white and shiny, indicating inflammation (Fig. 3A). The left renal vein was carefully dissected and the neck of the aneurysm, which was dilated to approximately 4 cm, was isolated. The external and internal iliac arteries on both sides were also isolated. After clamping each blood vessel without heparinization, the aneurysm was incised and a large intramural thrombus was removed, the vertebral body was exposed on the dorsal side of the aneurysm, confirming chronic rupture of the AAA (Fig. 3B). We performed vascular reconstruction with a tailor-made tapering graft [10] using a 22 × 11-mm Dacron bifurcated graft (Fig. 3C). The leg of the graft was anastomosed end-to-end to the right common iliac artery end and the left external iliac artery, and the left internal iliac artery was ligated. Blood loss was 988 ml, and no blood transfusion was required. The postoperative course was uneventful, and no change in the aortic dissection was observed. The patient was discharged home on the 13th postoperative day. The preoperative/postoperative characteristics of the patient are summarized in Table 1. After discharge, the diameter of the aorta was checked via CT every year, and it has slightly enlarged since 2 years after the surgery.

**Table 1:** Pre- and postoperative characteristics of the patient.

At the time of CT	At the time of first hospitalization	Two days after admission	Two weeks after admission	Preoperative	One year after operation	Two years after operation
Diameter of the ascending aorta	3.9 cm	3.7 cm	3.8 cm	4.1 cm	4.1 cm	4.0 cm
Location of thrombosis in the false lumen	A small portion of the ascending aorta	About halfway of the ascending aorta	From the ascending aorta to the aortic arch	From the ascending aorta to the aortic arch	From the ascending aorta to the aortic arch	From the ascending aorta to the aortic arch
Aortic diameter at entry site	3.9 cm	4.0 cm	4.1 cm	4.4 cm	4.7 cm	4.9 cm
Size of the AAA	11.5 × 9.8 cm	11.4 × 9.8 cm	11.4 × 9.9 cm	11.4 × 10.0 cm		

AAA, abdominal aortic aneurysm; CT, computed tomography.

## 3 Discussion

Giant AAAs have been reviewed in several papers [11,12], and they are rarely associated with acute aortic dissection. In case reports of acute TAAD with a giant AAA, the dissection and AAA were successfully treated concurrently or staged at short intervals [5,7]. However, concurrent surgery is extremely invasive and risky. In this case, conservative treatment was chosen for the TAAD. Retrograde TAAD with intramural hematoma is known to have a better prognosis than that of conventional TAAD [13,14]. For cases of retrograde TAAD with a patent false lumen in the ascending aorta, emergency surgery is recommended, as in conventional TAAD [15]. The prognosis of conservative treatment in these cases is currently unknown. Fortunately, the patient survived the acute phase, as the patient had a stable clinical history with few false intraluminal thrombi. However, whether conservative treatment would be successful in similar cases remains unknown. Therefore, if the dissection had progressed, emergency total aortic replacement would have been required. Due to the risk of progressive dissection from intraoperative aortic clamping during AAA surgery, it is desirable to perform AAA repair during the chronic stage of dissection rather than during the acute stage. In this case, AAA surgery did not worsen the dissection, and the patient was treated at an appropriate time; however, proper assessment of the respective conditions of the aortic dissection and AAA is necessary to determine the timing of treatment.

In this case, a giant AAA with CCR was identified. First reported by Jones et al. [16] in 1986, CCR is a condition in which a hematoma forms and enlarges in the retroperitoneal space due to repeated bleeding during the course of an aortic aneurysm rupture without major bleeding or shock. However, the membrane surrounding the hematoma eventually ruptures, and bleeding and rupture can occur again; therefore, surgical treatment should be performed early after diagnosis [17]. In our case, CCR of a giant AAA, which is an extremely dangerous condition, was detected. Although not possible in all cases of CCR, careful follow-up allowed us to determine the appropriate timing of treatment.

Open surgery was performed for the AAA repair in this case. For both giant AAA and CCR-AAA cases, there have been several reports of successful endovascular aneurysm repair (EVAR) [1,18,19]. Giant aneurysms are usually tortuous and associated with severe angulation of the proximal neck, rendering EVAR difficult [20]. In this case, EVAR would have likewise been difficult, as the patient had a juxtarenal AAA with a huge reverse tapering neck; therefore, graft reconstruction using a tailor-made tapering graft, which was previously reported by Shirasu et al. [10], was performed. This method is useful in surgery for AAAs with a large neck, where conventional EVAR would be difficult.

## 4 Conclusion

We reported the case of a giant AAA with a CCR diagnosed at the onset of acute TAAD. In cases of combined CCR of a giant AAA and retrograde TAAD, conservative management may be attempted to convert the acute dissection to a chronic one, thereby allowing elective repair of the AAA. Although the criteria are currently unclear, retrograde TAAD with a few false intraluminal thrombi can be treated conservatively. In complex cases of combined AAA and aortic dissection, determining the more critical lesion is necessary to formulate an individualized treatment plan for the patient.

## Patient consent statement

Written informed consent was obtained from the patient for the publication of this case report and accompanying images.

## Ethical approval

Permission from the ethics committee was not required for case reports at our institution.

## Funding

The authors declare that they received no funding for this work.

## Guarantor

Takafumi Akai will take full responsibility for the work.

## CRediT authorship contribution statement

Shintarou Ninomiya, MD: Involved in the conception and design of the study.
Takanori Kaneko: Involved in patient management, the conception and design of the study, and drafting and revising of the article.
Takatoshi Furuya: Involved in the conception and design of the study, final approval of the version to be submitted, and also involved in direct management of the patient.
Takafumi Akai: Involved in the design of the study, drafting and revising of the article, and final approval of the version to be submitted.

## Declaration of competing interest

The authors have no conflict of interest to declare.
